# Congenital Heart Defects in Pregnancies Conceived by Assisted Reproductive Technology: Comparing Functional and Structural Defects

**DOI:** 10.7759/cureus.54810

**Published:** 2024-02-24

**Authors:** Saeid Rasouli, Mohammad Radgoodarzi, Reza Azarbad, Azim Ghazvini, Mohammadjavad Sotoudeheian, Mehdi Taghizadeh, Mohammad Sedigh Dakkali

**Affiliations:** 1 Pediatrics, Iran University of Medical Sciences, Tehran, IRN; 2 Pediatric Cardiology, Iran University of Medical Sciences, Tehran, IRN; 3 Cardiology, Tabriz University of Medical Sciences, Tabriz, IRN

**Keywords:** ovulation induction, in vitro fertilization (ivf), fetal echocardiography, heart defects, congenital, birth defects, assisted reproductive technologies

## Abstract

Introduction

Congenital heart defects (CHD) are one of the most common congenital anomalies, and their association with assisted reproductive technology (ART) is controversial in different populations. The purpose of this study was to evaluate this association and to provide information about the necessity of specialized echocardiography during pregnancy with ART.

Methods

This retrospective study was performed on all pregnancies conceived by ART and referred for fetal echocardiography to the Rasoul Akram and Akbar Abadi hospitals in Tehran, Iran. A total of 109 patients were enrolled in the study (56 in the ART group and 53 in the non-ART). Two-dimensional and color Doppler echocardiography were performed on all patients to identify heart problems and anomalies and medical records of the patients were reviewed. The outcome was considered the presence of functional and structural heart defects on echocardiography.

Results

The study groups were similar in terms of maternal age and GA. The ART group consisted of 31 singletons (55%) and 25 multiples (45%). All pregnancies in the non-ART group were singletons. Following in vitro fertilization (33%), ovulation induction (25%) was the next most used method. The findings of echocardiography were one atrial septal defect (ASD) in ART and one in non-ART, six ventricular septal defects (VSD) in ART and three in non-ART, and one ASD and VSD in the ART group. These structural abnormalities showed no difference in the two groups (P value = 0.58). There was no significant difference in rhythm between the two groups (P = 0.51). Echocardiographic indices of both groups did not differ statistically except in the TR-PG index (P value = 0.02).

Conclusions

The structural defects of the two groups were not different, and no heart dysfunction was observed in ART fetuses. There was no association between ART and CHD in our study. We concluded that echocardiography by pediatric cardiologists is not necessary for these fetuses.

## Introduction

Congenital heart defects (CHD) are the most common cause of perinatal mortality associated with malformations [1]. It is six times more prevalent than chromosomal anomalies and four times more prevalent than neural tube defects among birth abnormalities [2,3]. CHD prevalence is estimated in up to 20 out of every 1,000 live births, and the incidence rate was 17.9/1,000 worldwide [4,5]. Inherited and non-inherited factors contribute to the risk of CHD. Less than 15% of CHD is caused by chromosomal problems or genetic anomalies, and, in most cases, the etiology is unknown [6]. Hence, assessing potential and controversial risk factors in different populations is crucial.

Assisted reproductive technology (ART) is widely used to treat infertility throughout the world, and it is a safe procedure that accounts for 1.6% of all births [7]. This method comprises different reproductive procedures, including in vitro fertilization (IVF), intrauterine insemination (IUI), ovulation induction (OI), and intracytoplasmic sperm injection (ICSI) [8]. ART is usually associated with favorable pregnancy outcomes; however, some reports indicate an increased risk of birth defects, especially heart defects in infants [8,9]. CHD events in ART pregnancies (1.3%) were higher than in non‐ART pregnancies (0.68%) [6]. ART-induced pregnancies were 1.37 times more likely to have mild cardiac anomalies than natural pregnancies [8]. Severe CHD occurred in 1.2% and 1.4% of natural pregnancies and ART pregnancies, respectively [10]. Other studies, however, found no association between ART and CHD, even when maternal age and multiple pregnancies were adjusted [11,12].

There is no consensus on whether ART should be considered an indication for fetal echocardiography, and there is no specific guideline for echocardiography for Iranian women [6]. To assess the necessity of echocardiography in this condition and to investigate the potential risk of CHD related to the usage of ART in the Iranian population, we conducted a study comparing ART-assisted and natural pregnancies.

## Materials and methods

Study design and population

This retrospective cohort study was conducted on all pregnancies conceived by ART referred to the Rasoul Akram and Akbar Abadi hospitals in Tehran, Iran, for fetal echocardiography between April 2019 to September 2020. ART included all treatments or procedures offered in clinics to women with infertility, such as ICSI, IUI, IVF, OI, and surrogacy. All patients referred for fetal echocardiography during the study period were included. Patients with the following medical conditions were excluded: hypertension, diabetes mellitus, heart problems and thyroid disorders, and systemic lupus erythematosus. Based on these criteria, 56 patients were involved in the ART group, and 53 patients were included in the non-ART group. The Iran University of Medical Sciences Ethics Committee approved this study (approval number: IR.IUMS.FMD.REC.1399.621).

Fetal echocardiography

The Phillips Affinity 70C system (Philips Affinity 70C, Andover, MA) was used to perform m-mode, two-dimensional, and conventional Doppler echocardiography on all of the pregnant women. This evaluation included a comprehensive functional and structural assessment. The cardiothoracic ratio, ejection fraction of ventricles, left-side cardiac output, diastolic filling period, and myocardial performance index were measured and calculated per the study by Dakkali et al. [13]. A single pediatric cardiologist performed all echocardiographic evaluations. The outcome was considered owing to the presence of functional and structural heart defects on echocardiography. Two groups were compared for echocardiographic indices, atrial septal defects (ASD), ventricular septal defects (VSD), valve regurgitation, and rhythm.

Data collection and analysis

Medical records were reviewed to gather demographic information and ultrasound results during pregnancy. A continuous variable was expressed by mean ± standard deviation or as a percentage (%) when appropriate. We used Student's t-tests for normal distributions and Mann-Whitney tests for non-normal distributions. Frequencies and percentages are presented for the categorical data. Statistical significance was determined by P values less than 0.05, and analysis of the data was conducted using Statistical Product and Service Solutions (SPSS) (version 18; IBM SPSS Statistics for Windows, Chicago, IL).

## Results

A total of 56 cases in the ART group and 54 cases in the non-ART group completed the study. Detailed demographic information about the study population can be found in Table 1. In terms of maternal age and GA, the study groups were similar (mean age in the ART group being 31.2 ± 6.8 and in the non-ART group being 28.83 ± 4.76, P value = 0.12; mean gestational age in the ART group being 25.86 ± 6.06 abd in the non-ART group being 25.42 ± 5.37, P value = 0.93).

**Table 1:** Demographic characteristics of the study population. min: minimum, max: maximum, GA: gestational age, ART: assisted reproductive technology, SD: standard deviation

Variables	ART (N= 56)	Non-ART (N= 53)	P value
Maternal age (mean ± SD)	31.2 ± 6.08	28.83 ± 4.76	0.12
Min age	22	22	
Max age	44	40	
GA (mean ± SD)	25.86 ± 6.06	25.42 ± 5.37	0.93
Min GA	18	18	
Max GA	40	37	
Number of pregnancies (mean ± SD)	2.02 ± 1.4	1.41 ± 0.69	0.03

The ART group consisted of 31 singletons (55%) and 25 multiples (45%). All pregnancies in the non-ART group were singletons. Most pregnancies were conceived using IVF (33%) and 25% through OI (Table 2). The method of ART was not recorded in two cases and is considered missing.

**Table 2:** Characteristics of pregnancies by ART. ICSI: intracytoplasmic sperm injection; IVF: in vitro fertilization, IUI: intrauterine injection, OI: ovulation induction, ART: assisted reproductive technology

Variables	N = 56
Singleton	31 (55%)
Multiple	25 (45%)
Type of ART	
ICSI	10 (17.8%)
IVF	19 (33.9%)
IUI	6 (10.7%)
OI	14 (25%)
Surrogacy	5 (8.9%)
Missed	2 (3.57%)

Based on the ultrasound results of the ART group, an increased nuchal translucency was reported in one case, along with seven cases of echogenic foci and two cases of choroid plexus cysts. Ultrasound in the non-ART group showed no pathological findings. There was a significant difference between the two groups (P value = 0.01).

Table 3 summarizes the echocardiography results in the study population. In the ART group, one ASD, one VSD, and one ASD and VSD were found. Echocardiography revealed one ASD and three VSD in the non-ART group. Analysis of structural abnormalities showed no difference (P value = 0.58). Additionally, the rhythms of the two groups were not significantly different (P = 0.51). Echocardiographic indices of ART and non-ART groups did not differ statistically except in the TR-PG index (Table 4, P value = 0.02).

**Table 3:** Structural findings of echocardiography in the study population. ART: assisted reproductive technology, ASD: atrial septal defects, VSD: ventricular septal defects, TR: tricuspid regurgitation

Variables	ART (N= 56)	Non-ART (N= 53)	P value
Structural findings			0.58
Normal	48	49	
ASD	1	1	
VSD	6	3	
ASD + VSD	1	0	
Rhythm			0.51
Normal	55	53	
Extrasystolic	1	0	
TR			0.19
Trivial	4	7	
Mild	9	0	
Moderate	1	0	

**Table 4:** Echocardiography indices in the study population. CT ratio: cardiothoracic ratio, LVEF: left ventricular ejection fraction, RVEF: right ventricular ejection fraction, RCOP: right-side cardiac output, LCOP: left-side cardiac output, LV-MPI: left ventricular myocardial performance index, RV-MPI: right myocardial performance index, TAPSE: tricuspid annular plane systolic excursion, MAPSE: mitral annular plane systolic excursion, MV E/A: mitral valve E-wave A-wave ratio, TV E/A: tricuspid valve E-wave A-wave ratio, LV-DFP: left ventricular diastolic filling period, RV-DFP: right ventricular diastolic filling period, TR-PG: tricuspid regurgitation peak gradient, SD; standard deviation. *These indices were nonparametric and analyzed with the Mann–Whitney test and represented as median and range

Index (mean ± SD)	ART	Non-ART	P value
CT ratio	0.44 ± 0.03	0.43 ± 0.02	0.77
LVEF	65.12 ± 3.05	64.75 ± 2.79	0.51
RVEF	68.3 ± 3.46	68.22 ± 3.31	0.82
RCOP*	709.8 ± 235.4	713.55 ± 241.56	0.96
LCOP	687.9 ± 220.03	675.4 ± 230.5	0.77
LV-MPI	0.42 ± 0.02	0.42 ± 0.02	0.5
RV-MPI	0.35 ± 0.02	0.36 ± 0.02	0.08
TAPSE*	0.44 ± 0.06	0.43 ± 0.06	0.23
MAPSE	0.46 ± 0.05	0.46 ± 0.05	0.92
MV E/A*	0.61 ± 0.13	0.61 ± 0.01	0.18
TV E/A*	0.61 ± 0.01	0.61 ± 0.01	0.6
LV-DFP	40.2 ± 0.71	40.67 ± 0.85	0.4
RV-DFP	40.34 ± 0.84	40.42 ± 1.04	0.52
TR-PG*	1.11 ± 0.8	0.4 ± 0.01	0.02

In the ART group, there were no significant differences in pathological findings with cardiac septal defects (P value = 0.61). The type of ART used did not significantly influence cardiac septal defects (Table 5, P value = 0.4). All septal defects were observed in singleton pregnancies, and the two groups differed significantly (P value = 0.01).

**Table 5:** Structural defects based on the type of ART. ICSI: intracytoplasmic sperm injection, IVF: in vitro fertilization, IUI: intrauterine injection, OI: ovulation induction

ART type	Structure		Total	P value
	Heart defect	Normal		
Missing	0	2	2	
ICSI	0	10	10	
IUI	1	5	6	
IVF	5	14	19	
OI	2	12	14	
Surrogacy	0	5	5	
Total	8	48	56	0.403

## Discussion

According to our findings, ART-conceived pregnancies are not at higher risk of CHD than natural pregnancies. The prevalence of septal defects was 14.2% in the ART group and 7.5% in the non-ART group, without statistical significance. Simpson et al. [14] concluded that the risk of major birth defects including anomalies of the heart is not increased in pregnancies with ART. Our finding is consistent with this study and the previous report.

It is controversial whether singleton or multiple pregnancies are associated with heart defects. A study by Wen et al. [15] demonstrated a higher risk of CHD in multiple pregnancies (OR: 1.68, CI: 1.48-1.95). After adjusting the results, this risk decreases between the ART group and the reference population. Reefhuis et al. [16] showed that septal defects are more likely to occur in singleton pregnancies with ART (OR: 2.1, CI: 1.1-4), and multiple pregnancies were not associated with birth defects after matching. According to our findings, structural defects were significantly higher in singleton pregnancies.

A common finding in fetal echocardiography is tricuspid valve regurgitation, which is usually considered benign alongside normal heart structure. The condition is found in 6.8-83.4% of pregnancies, and it is characterized by a regurgitation of blood into the right atrium during a right ventricular contraction [17,18]. Our study found a prevalence of 25.2% in the ART group and 18.8% in the non-ART group without statistical significance. In our study, TR is a benign finding in pregnancies with ART, similar to the natural population, and does not indicate a cardiac abnormality.

During prenatal sonography, soft markers can be used to predict abnormalities [19]. If more than one soft marker is observed during sonography, a more accurate sonography should be conducted, and, if the results are confirmed, an amniocentesis should be performed [20]. In our study, soft markers were isolated findings without other pathological features or associations with heart disease.

## Conclusions

In our study, ART fetuses have similar structural and functional heart development compared with natural pregnancies, and they are not at higher risk of CHD. Hence, we conclude that ART fetuses under perinatologist care do not require specialized echocardiography during the fetal period, and ultrasound examinations may effectively assess heart function and structure. However, our study had some limitations. We considered fetal echocardiography, but some heart defects, such as patent ductus arteriosus (PDA) and VSD, require echocardiography after birth. This study evaluated structural wall defects of the heart that are more common. In the future, larger sample sizes will be used to identify other major heart defects, such as tetralogy of Fallot, coarctation of aorta, and so on. Additionally, COVID-19 caused limitations in data collection, which led to a smaller sample size.
